# Fast electrodeposition of zinc onto single zinc nanoparticles

**DOI:** 10.1007/s10008-020-04539-9

**Published:** 2020-03-14

**Authors:** Giorgia Zampardi, Richard G. Compton

**Affiliations:** 1 Department of Chemistry, Physical and Theoretical Chemistry Laboratory, University of Oxford, South Parks Road, Oxford, OX1 3QZ UK; 2 Present Address: Universität Bremen, Energiespeicher- und Energiewandlersysteme, Bibliothekstraße 1, 28359 Bremen, Germany

## Abstract

The zinc deposition reaction onto metallic zinc has been investigated at the single particle level through the electrode-particle collision method in neutral solutions, and in respect of its dependence on the applied potential and the ionic strength of a sulphate-containing solution. Depending on the concentration of sulphate ions in solution, different amounts of metallic zinc were deposited on the single Zn nanoparticles. Specifically, insights into the electron transfer kinetics at the single particles were obtained, indicating an electrically early reactant-like transition state, which is consistent with the rate-determining partial de-hydration/de-complexation process. Such information on the reaction kinetics at the nanoscale is of vital importance for the development of more efficient and long-lasting nanostructured Zn-based negative electrodes for Zn-ion battery applications.

**Electronic supplementary material:**

The online version of this article (10.1007/s10008-020-04539-9) contains supplementary material, which is available to authorized users.

## Introduction

The growing need of our contemporary society for energy harvested from renewable sources is pushing the development of energy storage systems for large-scale stationary applications. Considering their high costs, the toxicity, and the safety issues of the organic-based electrolytes, together with the uneven distribution of the lithium resources [1–3], Li-ion batteries are not ideal candidates for stationary applications. For this reason, in the last decade, efforts have been directed towards the development of aqueous-based metal-ion batteries, because of the more environmentally friendly nature of the electrode materials and of the electrolytes constituting the cell, and because of their lower costs. Aqueous metal-ion batteries are based on monovalent Na-ion and K-ion, or on polyvalent Zn-ion, Al-ion, and Mg-ion insertion chemistries [4–6]. In contrast to the more mature Li-ion concept, all these technologies to be commercialised need still advancement in terms of increasing efficiencies and cycle life, and electrode material optimisation [4, 6, 7]. Among the different aqueous metal-ion technologies, Zn-ion batteries consisting of a metallic Zn-based negative electrode, a Zn^2+^-containing aqueous electrolyte, and a Zn insertion positive electrode are attracting a great interest within the scientific community. This is due to the low cost of metallic zinc, being an earth-abundant element, to its high volumetric and gravimetric capacity (of 5855 mAh cm^−3^ and of 820 mAh g^−1^, respectively), and to its low standard reduction potential (− 0.76 V vs. SHE), which still allows the operation of the battery in aqueous electrolytes [5, 8]. Despite the fact that the application of aqueous Zn-ion batteries is mostly limited by the lack of efficient Zn insertion materials with long cycle life, to be used in the positive electrode side [5], many limitations still need to be overcome on the negative electrode side as well [9–11]. The major problems yet to be solved involving the metallic zinc electrode are related to the hydrogen evolution reaction occurring in parallel to the Zn deposition reaction [9–11], and the non-uniform deposition of metallic Zn leading to the formation of dendrites, and consequently to short-circuit of the electrodes [11]. Despite the fact that the zinc deposition and dissolution reaction has been studied for decades, mainly for corrosion and electrodeposition applications [12–17], further investigations are yet needed regarding the Zn reaction mechanisms in the solutions employed within the Zn-ion batteries. Several studies have shown that the reaction mechanism of the zinc deposition and dissolution reaction differs strongly in the dependence on the electrolyte in terms of the type of solvent (i.e., organic or water based), pH, anions, cations, complexing agents, additives, etc. [18]. Moreover, the negative electrodes that are used in Zn-ion batteries are generally constituted of a Zn foil [19], a Zn deposit on a substrate [20], or a composite electrode with Zn particles [10, 21]. Considering the Zn atomic weight of 65.39, an ideal negative electrode of a Zn-ion battery would be constituted of a low amount of metallic Zn, which can be efficiently and reversibly used, in order not to decrease the gravimetric energy and power density of the final battery. For this reason, research efforts are directed towards the fabrication of nanostructured electrodes employing Zn nanoparticles embedded in a substrate [22]. This makes necessary the study of the kinetics of the Zn deposition/dissolution reaction at the nanoscale as well, since the electrochemical behaviour of a material may differ at the nanoscale compared with the one at the macroscopic level [23]. In this respect, the electrode-particle collision method (often referred to as ‘nanoimpacts method’) has been shown to give important insights into the electrochemical behaviour in general, and the reaction kinetics in particular, of a variety of materials at the single particle level [24–33]. This method consists in dispersing a small amount of particles in solution, which, by virtue of their Brownian motion, may collide with the surface of a microelectrode. The microelectrode is polarised at a potential such that the stochastic collisions of the nanoparticles result in spikes in the background current recorded at the microelectrode [26, 33–38]. The magnitude of the charge, duration, and frequency of a numerically relevant number of single collision events can be analysed statistically in order to harvest information on the reactions occurring at the nanoscale [28–30, 32, 38, 39]. Electrode-particle collision experiments have been employed to analyse the electrochemical behaviour and the reaction kinetics of a variety of systems: from metal deposition [27] and dissolution [40], to ion (de-)insertion reactions [23, 41–43].

In this paper, the zinc deposition reaction onto metallic Zn has been investigated at the single particle level through the electrode-particle collision method in neutral solutions containing sulphate ions, leading to information on the electron transfer kinetics at the nanoscale.

## Experimental

### Materials and characterisation

Zinc particles (zinc nanopowder, 40–60-nm nominal average particle size), zinc sulphate, and potassium sulphate were obtained from Sigma-Aldrich, UK, and used as received. All the solutions were prepared using ultrapure water (Millipore) with a resistivity of 18.2 MΩ cm at 298 K.

Scanning electron microscopy (SEM) imaging was performed with a JEOL JSM-6500F scanning electron microscope with an accelerating voltage of 5 kV. The samples were prepared through the drop-cast of zinc nanoparticles from a solution containing 0.05 g L^−1^ of Zn nanoparticles (NPs) on a conductive glassy carbon substrate, followed by drying under a nitrogen atmosphere. The Zn NPs were dispersed following the same procedure employed to suspend the nanoparticles prior to the electrode-particle collision experiments (see below). Prior to the Zn NP drop-cast, the glassy carbon plate was treated with aqua regia in order to provide a clean surface and rinsed with ultrapure deionised water.

Dynamic light scattering (DLS) measurements were performed with a Malvern Zetasizer Nano ZS instrument in order to measure the hydrodynamic diameter of the Zn NPs when dispersed in solution. The samples consisted in a suspension of Zn NPs with a concentration of 0.1 g L^−1^ in ultrapure deionised water, placed in a disposable solvent-resistant micro-cuvette with a path length of 10 mm.

### Electrode-collision experiments and cyclic voltammetry experiments

Both collision and cyclic voltammetry experiments were performed in a three-electrode electrochemical cell put inside a double Faraday cage. An in-house-built low-noise potentiostat was used with a 100-Hz Bessel-type low-pass filter [44]. The analog-to-digital and digital-to-analog conversion was provided by a USB-6003 DAQ (National Instruments, TX, USA). These devices were controlled through a script (Python 2.7) with a graphical user interface and real-time electrochemical data visualization based upon the packages provided in the Enthought Tool Suite (Enthought, TX, USA). Note that the potentiostat system used is designed to accurately conserve the charge even if the spike shapes are distorted at short (millisecond) timescales. Pt foil (Goodfellow, UK) and a saturated calomel electrode (SCE, + 0.241 V vs. standard hydrogen electrode, SHE) were used as the counter and the reference electrodes, respectively, and a carbon microdisc electrode with a 33-μm diameter (ASL, Japan) was used as working electrode. Prior to each experiment, all the solutions were vigorously purged with nitrogen in order to reduce the amount of dissolved oxygen.

In order to homogeneously suspend the zinc nanoparticles, a VCX400 sonic horn (Sonics and Materials, USA) with a maximal power of 400 W was used with a 3-mm titanium alloy probe. Small volumes (2 mL) of solutions containing the zinc nanoparticles were sonicated at 13% of the maximum power amplitude with a pulse mode (3 s ON and 3 s OFF) for 30 s. When the experiment required a suspension of Zn NPs, the solution was initially purged with nitrogen and then sonicated with the sonic horn.

## Results and discussion

The zinc deposition reaction was initially studied via cyclic voltammetry, with the deposition occurring onto a carbon microdisc electrode immersed in an unsupported aqueous neutral solution containing 1 mM ZnSO_4_. The reaction occurring at the carbon microelectrode is the following:

$$ {\mathrm{Zn}}^{2+}+2{\mathrm{e}}^{-}\rightleftharpoons {\mathrm{Zn}}^0 $$

**Fig. 1:**
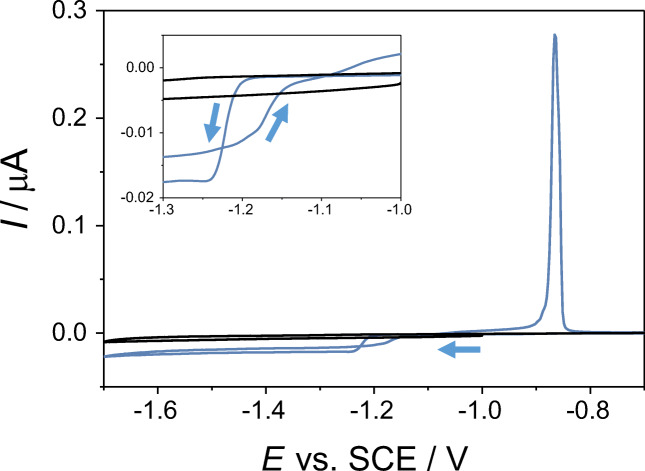
Cyclic voltammetry of a carbon microdisc electrode with a diameter of 33 μm in a solution containing 1 mM ZnSO_4_ (blue line) and in a solution containing 1 mM K_2_SO_4_ (black line). The scan rate was 25 mV s^−1^. The inset shows the starting potential of the deposition of metallic zinc from the Zn^2+^-containing solution (c.a. − 1.2 V vs. SCE)

Starting from the open-circuit potential (c.a. − 1 V vs. SCE), the carbon microelectrode was polarised cathodically at 25 mV s^−1^ until reaching a potential of − 1.7 V vs. SCE. In Fig. 1, the Zn deposition starts at a potential of c.a. − 1.2 V vs. SCE, in agreement with that of the literature [10]. The rather flat cathodic current observed for potentials more negative than − 1.25 V vs. SCE is related to the continuous deposition of zinc from the solution onto the electrode surface, and in a smaller extent to the hydrogen reduction, whose overpotential is high at carbon as well as zinc substrates [10, 45, 46]. Upon oxidation, two typical crossover points between the anodic and the cathodic branches of the cyclic voltammetry can be observed at potentials of c.a. − 1.22 V and of − 1.1 V vs. SCE. The crossover point observed at more cathodic potentials (in this case − 1.22 V vs. SCE) is usually ascribed to the nucleation of the new phase formed upon the zinc deposition [45]. Proceeding along the anodic branch of the voltammetry, the metallic zinc previously deposited onto the carbon microelectrode is oxidised at a potential of c.a. − 0.9 V vs. SCE.

Next, the reaction was investigated at the single particle level through the electrode-particle collision method. Zn nanoparticles (NPs) were dispersed in a solution containing zinc ions and a carbon microdisc electrode, which was polarised at a suitable potential in order to deposit zinc from the solution onto the Zn particle. When 44 pM of Zn NPs was dispersed in solution, clear reductive spikes appeared in the baseline current of the chronoamperograms recorded at the carbon microelectrode. Such spikes are related to the deposition of Zn onto the Zn nanoparticles that stochastically come in contact with the surface of the microelectrode, according to the reaction:

$$ {\mathrm{Zn}}^{2+}+2{\mathrm{e}}^{-}\to \kern0.5em {\mathrm{Zn}}^0 $$

The electrode-particle collision experiments were performed at different potentials applied at the microelectrode, with different concentrations of dispersed Zn NPs, and in neutral electrolytic solutions having different ionic strengths, leading to consideration of the reaction kinetics of the zinc deposition occurring at the nanoscale.

Reductive spikes were observed at potentials negative of − 1.4 V vs. SCE in a solution containing 1 mM ZnSO_4_. An overpotential of c.a. − 0.2 V was thus required to allow the reaction to occur at the nanoparticle scale. Hence, the electrode-particle collision experiments were recorded at − 1.4 V, − 1.5 V, − 1.6 V, and − 1.7 V vs. SCE. At all these applied potentials, the background current recorded at the microelectrode had a Faradaic component, besides a possible capacitive one, due to the concomitant deposition of Zn^2+^ from the solution onto the microelectrode surface. In order to ensure that the spikes recorded during the chronoamperograms in presence of the Zn NPs truly corresponded to the deposition of zinc *onto the single nanoparticles* colliding against the microelectrode surface, three types of control experiments were performed.

**Fig. 2:**
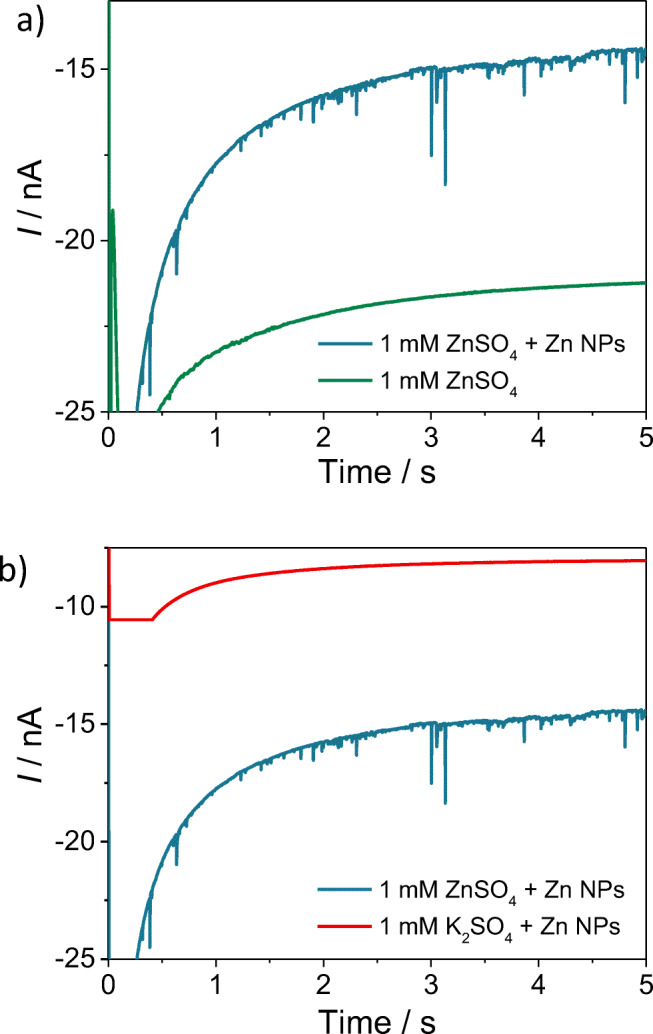
**a** Chronoamperograms of a carbon disc microelectrode immersed in a solution containing 1 mM ZnSO_4_ with (blue line) and without (green line) 44-pM Zn nanoparticles. **b** Chronoamperogram of a carbon disc microelectrode immersed in a solution containing 44-pM Zn nanoparticles and 1 mM ZnSO_4_ (blue line) or 1 mM K_2_SO_4_ (red line). In both **a** and **b**, the potential applied to the microelectrode was − 1.6 V vs. SCE

First, chronoamperograms were recorded at the carbon microelectrode immersed in a solution containing 1 mM ZnSO_4_ (without Zn NPs in solution). In this case, as shown in Fig. 2a, no reductive spikes were observed. In this way, it was demonstrated that the reductive spikes did not arise from the presence of noise in the Faradaic baseline current deriving from the zinc deposition reaction onto the carbon microelectrode.

Secondly, chronoamperograms were performed at the carbon microelectrode immersed in a solution containing 1 mM K_2_SO_4_ and 44 pM of Zn NPs (without Zn^2+^ cations in solution). Also in this case, as shown in Fig. 2b, no reductive spikes were observed. The fact that the Zn NPs in solution colliding against the microelectrode did not produce any spike in absence of Zn^2+^ cations, demonstrated that, when observed, the reductive spikes do not originate from a local hydrogen evolution that may occur at the surface of the Zn NP [10, 46]. When the electrode is polarised at high cathodic overpotentials, the recorded current at the macroscopic Zn-based electrode is ascribed, at least to some small extent, to the hydrogen evolution reaction even in neutral solutions [10, 46]. Nevertheless at the nanoscale, the lack of reductive features in absence of Zn^2+^ cations suggests that no significant amount of hydrogen is produced at the single Zn particles during the collision experiments.

This control experiment furthermore demonstrates that the reductive spikes, when recorded, are also not related to the reduction of the thin oxidation layer that is likely to be present onto the surface of the metallic Zn NPs. Such a layer is likely to originate from the initial exposure of the Zn nanoparticles to air and moisture. However, it does not further grow significantly when the Zn particles are in contact with air and water, as demonstrated by the experimental findings of Funke et al. [47], and by the low zinc corrosion rates [13, 15, 16], considering the short time frame of the electrode-particle experiments (c.a. 5 min).

Both types of control experiments were performed for all the potentials applied during the electrode-particles collision measurements, as shown within the Supporting Information (Section 1 Figure SI_1, and Section 2 Figure SI_2).

A third kind of control experiment was performed with the carbon microelectrode immersed in a solution containing 44 pM of Zn NPs and 1 mM ZnSO_4_. In this case, potentials ranging from − 1.1 to + 1 V vs. SCE have been applied at the microelectrode, as shown in the Supporting Information (Section 3, Figure SI_3). Since for potentials more positive than − 1.2 V vs. SCE no zinc deposition is supposed to occur, the fact that no reductive spikes in the background current have been detected further demonstrates that, when observed, the transient spikes are not related to the capacitive current, but to the actual deposition of Zn^2+^ onto the Zn nanoparticle.

A Zn particle average size of c.a. 500 nm was estimated through the dynamic light scattering (DLS) analysis of a suspension of Zn nanoparticles in ultrapure water. Such estimated diameter is considerably higher than the nominal one indicated by the supplier (i.e., 40–60 nm), thus indicating particle agglomeration/aggregation when suspended in solution. This was further confirmed by the scanning electron microscopy (SEM) imaging of a 0.05 g L^−1^ suspension of Zn nanoparticles drop-cast onto a glassy carbon electrode and dried under a flow of nitrogen (Supporting Information Section 4 Figure SI_4), where the Zn nanoparticles appear to be agglomerates/aggregates of smaller particles with an irregular shape. Such observed significant clustering is likely due to agglomeration/aggregation of the particles upon their suspension in solution and upon drying. It is worth noticing that in spite of the agglomerate/aggregate nature of the suspended Zn particles, we will consider them as having a regular spherical shape for the sake of an easier calculation of the average parameters obtained during the collision experiments.

**Fig. 3:**
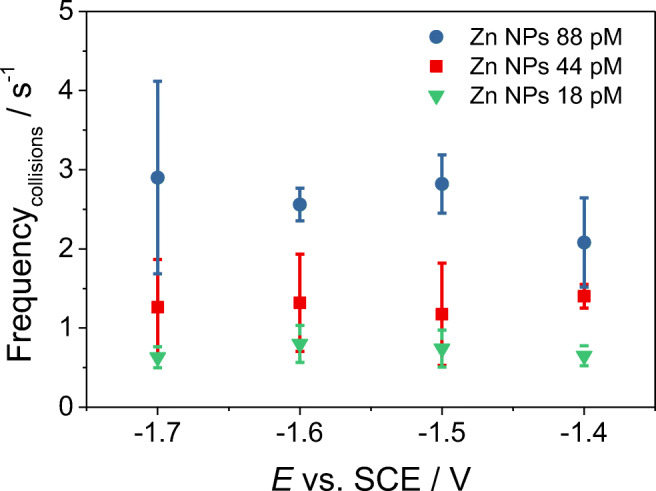
Average frequency of the collision events measured at different potentials applied at the carbon microelectrode immersed in a solution containing 1 mM ZnSO_4_, with different concentrations of dispersed Zn nanoparticles. The average collision frequency was calculated by taking into account also the chronoamperograms recorded in presence of the dispersed Zn NPs displaying a low number of collision events

The average frequency of the reductive events recorded in a solution containing 1 mM ZnSO_4_ is shown in Fig. 3, and it was found to be independent of the potentials applied to the microelectrode, whilst as expected, it was dependent on the concentration of Zn nanoparticles present in solution. From the Stokes-Einstein equation and considering the average particle diameter of 500 nm, the average diffusion coefficient of a single Zn nanoparticle was estimated to be c.a. 9.8 × 10^−13^ m^2^ s^−1^. The average collision frequency was then predicted using the average particle diffusion coefficient, and an integrated form of the Shoup-Szabo equation. In particular, this was calculated to be c.a. 2.3 s^−1^, 3.5 s^−1^, and 4.6 s^−1^ in 1 mM ZnSO_4_ solutions containing 18 pM, 44 pM, and 88 pM of Zn nanoparticles, respectively. As it can be seen in Fig. 3, the experimental collision frequencies, despite increasing with increasing concentrations of Zn NPs in solution, are a little lower than the estimated theoretical ones, which take into account a diffusion-only mass transport of the particles, and neglect the hindered diffusion processes occurring in very close proximity to the interface [48, 49]. The little discrepancy between the experimental and the predicted values of the collision frequencies may as well result from errors in the estimation of the average particle diameter, which has been considered as a perfect sphere. According to the DLS and the SEM measurements, once suspended in solution, the Zn NPs are most likely to have an irregular shape, influencing the calculation of the particles diffusion coefficients and therefore their estimated collision frequencies. The experimental average collision frequency was calculated by taking into account also the chronoamperograms recorded in presence of Zn NPs displaying a low number of collision events. In some cases, the experimental collision frequency in the recorded chronoamperogram appears higher than the theoretical one (as in Fig. 2, SI_1, and SI_2). This can be explained in view of the fact that in the calculation of the theoretical collision frequency, the smaller agglomerates/aggregates, which are most likely to be present in solution, are not taken into account. It is worth noticing that the average charge obtained during the collision experiments measured at different potentials applied at the microelectrode was found to be independent from the amount of Zn nanoparticles immersed in solution (see Supporting Information Section 5 Figure SI_5).

The average duration of the reductive spikes was determined through their full width half maximum (FWHM), namely the width of an individual reductive event at half of the maximum of its current amplitude, and it was found to be constant for all the potentials applied to the microelectrode, and for all the concentrations of Zn nanoparticles. In particular, in 1 mM ZnSO_4_ solutions containing 18 pM, 44 pM, and 88 pM of Zn nanoparticles, the average collision duration was 6.6 ± 1.4 ms, 8.3 ± 1.2 ms, and 7.3 ± 1.6 ms, respectively (as shown in the Supporting Information Section 6 Figure SI_6). These values likely reflect the response time of the potentiostat system used.

It is worth noticing that the experimental observation of a non-continuous reductive current during the electrodeposition of Zn onto Zn nanoparticles (i.e., on-off behaviour) is related to the contact time between the Zn NPs and the microelectrode, therefore to the intrinsic nature of the collision experiment itself.

**Fig. 4:**
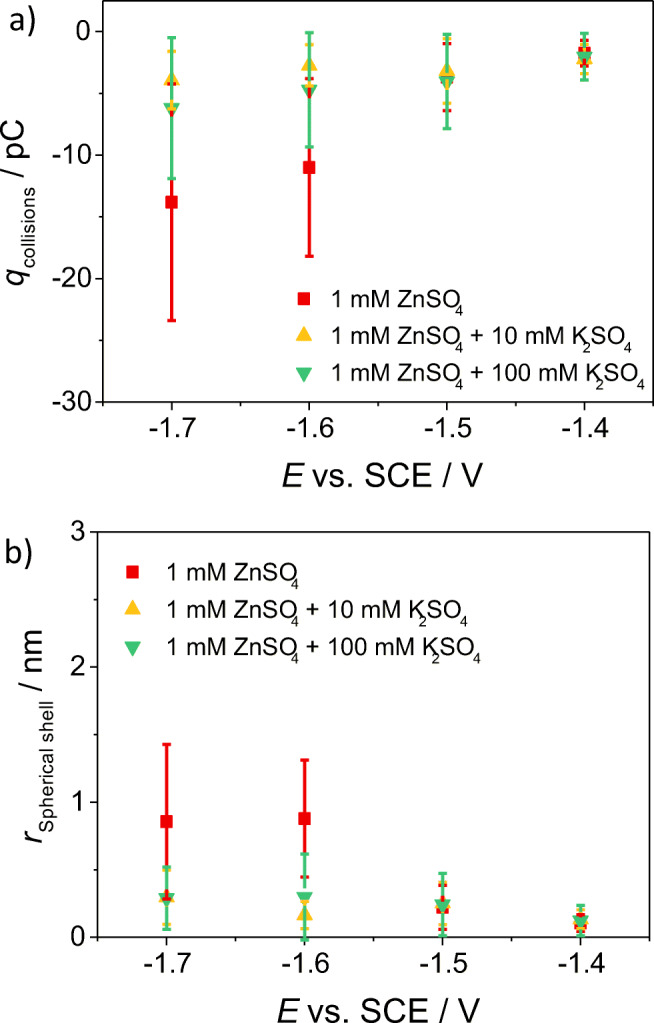
**a** Average charge of the collision events and **b** radius of the spherical shell of the zinc deposited onto the single particle upon collision, recorded at different potentials applied at the carbon microelectrode immersed in a solution containing 44 pM of Zn nanoparticles and in three different 1 mM ZnSO_4_–containing solutions

The zinc deposition at the nanoscale was investigated also in terms of its dependence on the ionic strength of the solution. Therefore, electrode-particle collision experiments were also recorded in solutions containing 10 mM and 100 mM K_2_SO_4_ as supporting electrolyte. Both the average collision frequency and the average collision duration remained constant with the potential applied at the microelectrode, when the experiments were recorded in the supported solutions. Moreover, they followed the same trend of the average frequency and duration of the collisions recorded in the unsupported 1 mM ZnSO_4_ solution (as shown in the Supporting Information Section 7 Figure SI_7, and SI_8). From Fig. 4a, the average reductive charge of the collision events increased as the microelectrode was polarised more cathodically in the unsupported 1 mM ZnSO_4_ solution. On the other hand, the average reductive charge recorded in the supported solutions with both 10 mM and 100 mM K_2_SO_4_ increased relatively slightly upon changing the electrode potential. However, in all cases, the potential dependence is weak.

Assuming that the zinc deposition occurred homogenously at a single spherical Zn particle colliding against the microelectrode, the radius of the spherical shell of the zinc deposited onto the single spherical particle was calculated. As shown in Fig. 4b, considering a Zn nanoparticle with an average diameter of 500 nm, the spherical shell radius increased by an amount between c.a. 0.25 and c.a. 1 nm in the unsupported 1 mM ZnSO_4_ electrolyte, when the electrode potential was changed from − 1.5 to − 1.6 V vs. SCE. In presence of the supporting electrolyte, the radius of the zinc shell deposited onto the average Zn nanoparticle remained around c.a. 0.25 nm for all the potentials applied at the microelectrode.

The smaller amount of reductive charge (and consequently the smaller amount of zinc deposited onto the Zn NPs) recorded during the collision experiments in the supported solutions containing 10 mM and 100 mM K_2_SO_4_ can be explained considering two effects: (I) a higher charge screening effect of the supporting electrolyte towards the Zn NPs and (II) a higher amount of SO_4_^2−^ anions in solution. Sulphate ions tend to hinder the zinc deposition reaction, because of the occurrence of a strong ion pairing between the Zn^2+^ cations and the SO_4_^2−^ anions; stable sulphate complexes are thus formed, with a consequent hindered release of the Zn^2+^ cations, leading to a lower concentration of free Zn^2+^ in solution [45, 50].

**Fig. 5:**
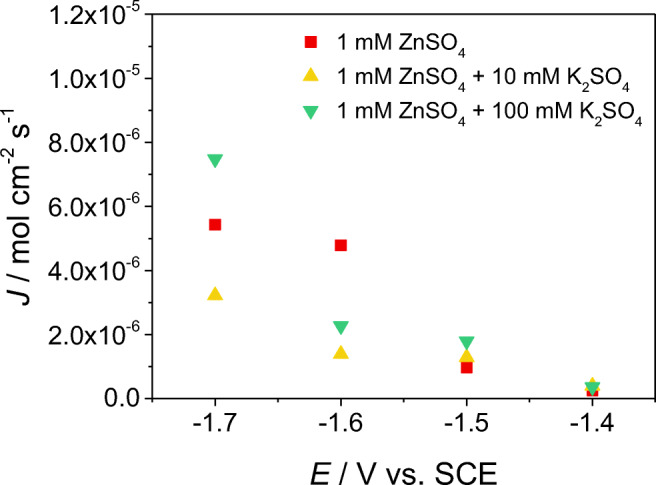
Estimated flux of the Zn deposition reaction occurring at the single nanoparticle, in dependence on the potential applied to the microelectrode, in three different 1 mM ZnSO_4_–containing solutions

Last, we considered the kinetics of the electron transfer upon zinc deposition onto the single colliding Zn nanoparticles. Figure 5 shows a plot of the flux of zinc deposition calculated considering an average Zn nanoparticle with a diameter of 500 nm, the average current recorded during the collision events, and the measured duration of the spikes. Whilst in all cases, both supported and unsupported electrolytes, the flux increases with the electrode potential, its dependency is far from the exponential response typically expected for a system displaying Butler-Volmer kinetics. This may be due, at least in part, to the loss of ‘driving force’ due to the size of the diffuse layer, the so-called Frumkin corrections, but this is unlikely to play a significant role in the presence of 100 mM K_2_SO_4_.

A cathodic transfer coefficient was estimated from the high support data and found to be c.a. 0.17, suggesting that the first electron transfer in the overall two-electron process is rate determining. Moreover, such an exceptionally low value indicates that the transition state is reactant-like in terms of electrical charge. This in turn suggests that the activation energy of the process is significantly controlled by a partial de-hydration and/or de-complexation of the reacting cation prior to the electron transfer. Furthermore, if an estimate of the standard rate constant for the process is made assuming a formal potential of − 1.003 V (vs. SCE) [51], this is found to be of the order of 2 cm s^−1^ (see Supporting Information Section 8), indicating a very fast process.

## Conclusions

The zinc deposition reaction in neutral aqueous solutions has been investigated at the nanoscale through the electrode-particle collision method. It has been demonstrated that the clear reductive spikes recorded were due to the deposition of zinc onto the Zn nanoparticles that stochastically collided with the microelectrode surface, and they were related neither to the hydrogen evolution reaction nor to the reduction of the thin oxide layer that is likely to be present onto the metallic zinc nanoparticles. It has been observed that at the nanoscale, the extent of the zinc deposition reaction increased with increasing applied cathodic overpotential and decreased with increasing amount of sulphate in solution. The latter point is most likely due to the formation of stable zinc-sulphates complexes, which thus reduce the amount of free Zn^2+^ in solution. The kinetics of the electrodeposition and their (weak) potential dependence suggest that de-complexation and/or de-hydration step plays a significant role in controlling the rate of the reaction. Despite the zinc deposition/dissolution reaction having been studied for many years mainly for corrosion and electrodeposition applications [12–17], further studies of the reaction kinetics are needed in the solutions that are to be employed in Zn-ion batteries. Moreover, particular attention should be given to the reaction kinetics studies at the nanoscale, since nanocomposite and nanostructured Zn-based electrodes are very promising for the development of stable, efficient, and long-lasting negative electrodes for aqueous Zn-ion batteries.

## Electronic supplementary material

ESM 1 (PDF 1160 kb)
