# Supplementary material for: Fast electrodeposition of zinc onto single zinc nanoparticles
Source: J Solid State Electrochem. 2020 Mar 14;24(11):2695–702. doi: 10.1007/s10008-020-04539-9 (PMC7561586; doi:10.1007/s10008-020-04539-9)
Supplement: Supplementary file 1 — (PDF 1160 kb) [file 10008_2020_4539_MOESM1_ESM.pdf]

# Supporting Information

## **Fast Electro-Deposition of Zinc onto Single Zinc Nanoparticles**

Giorgia Zampardi §, and Richard G. Compton *

Department of Chemistry, Physical and Theoretical Chemistry Laboratory, Oxford University, South Parks Road, Oxford, OX1 3QZ, United Kingdom

§ Present Address: Universität Bremen, Energiespeicher- und Energiewandlersysteme, Bibliothekstraße 1, 28359 Bremen, Germany

1. **Control Chronoamperograms: with and without Zn Nanoparticles in Solution**

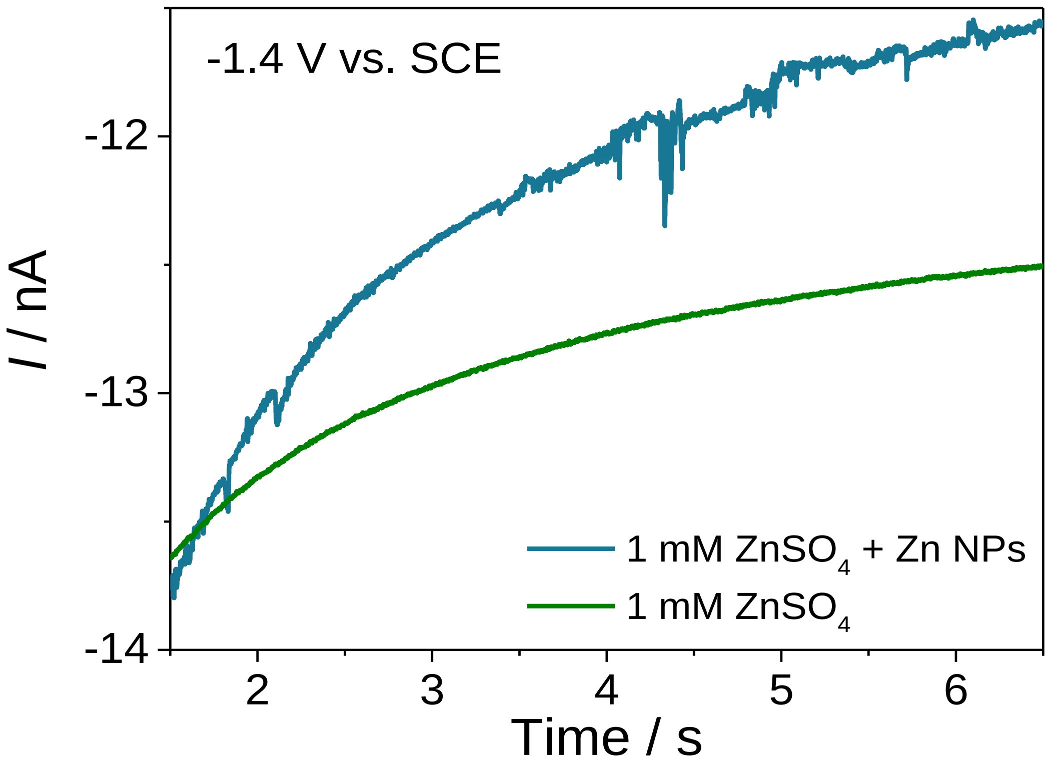


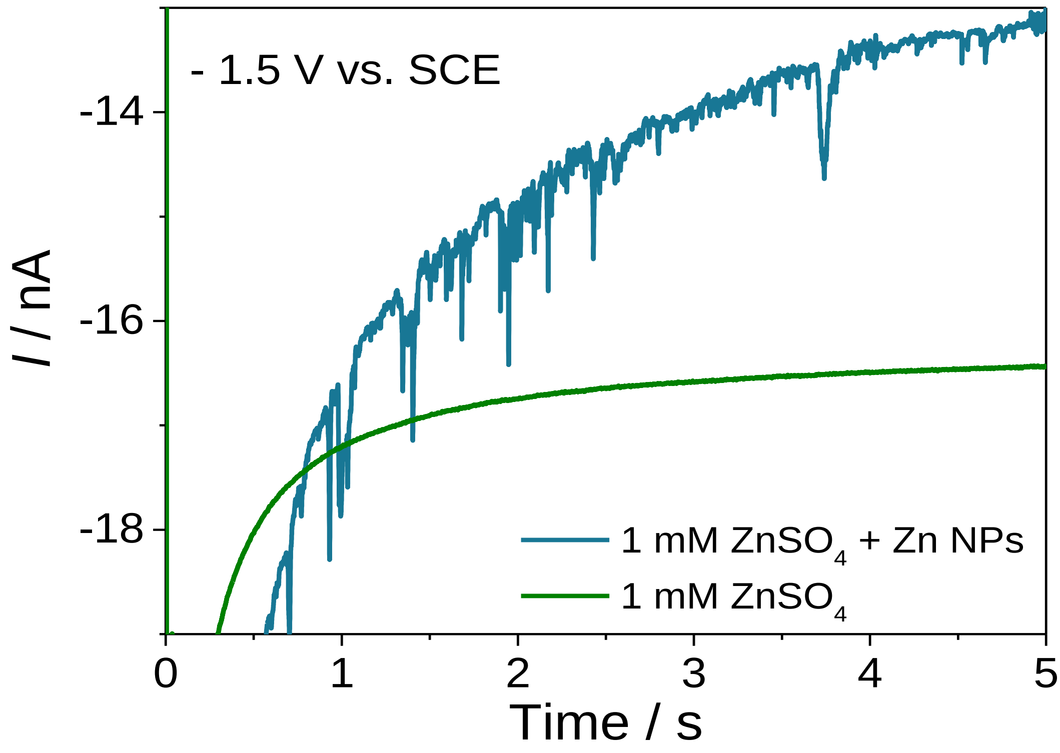

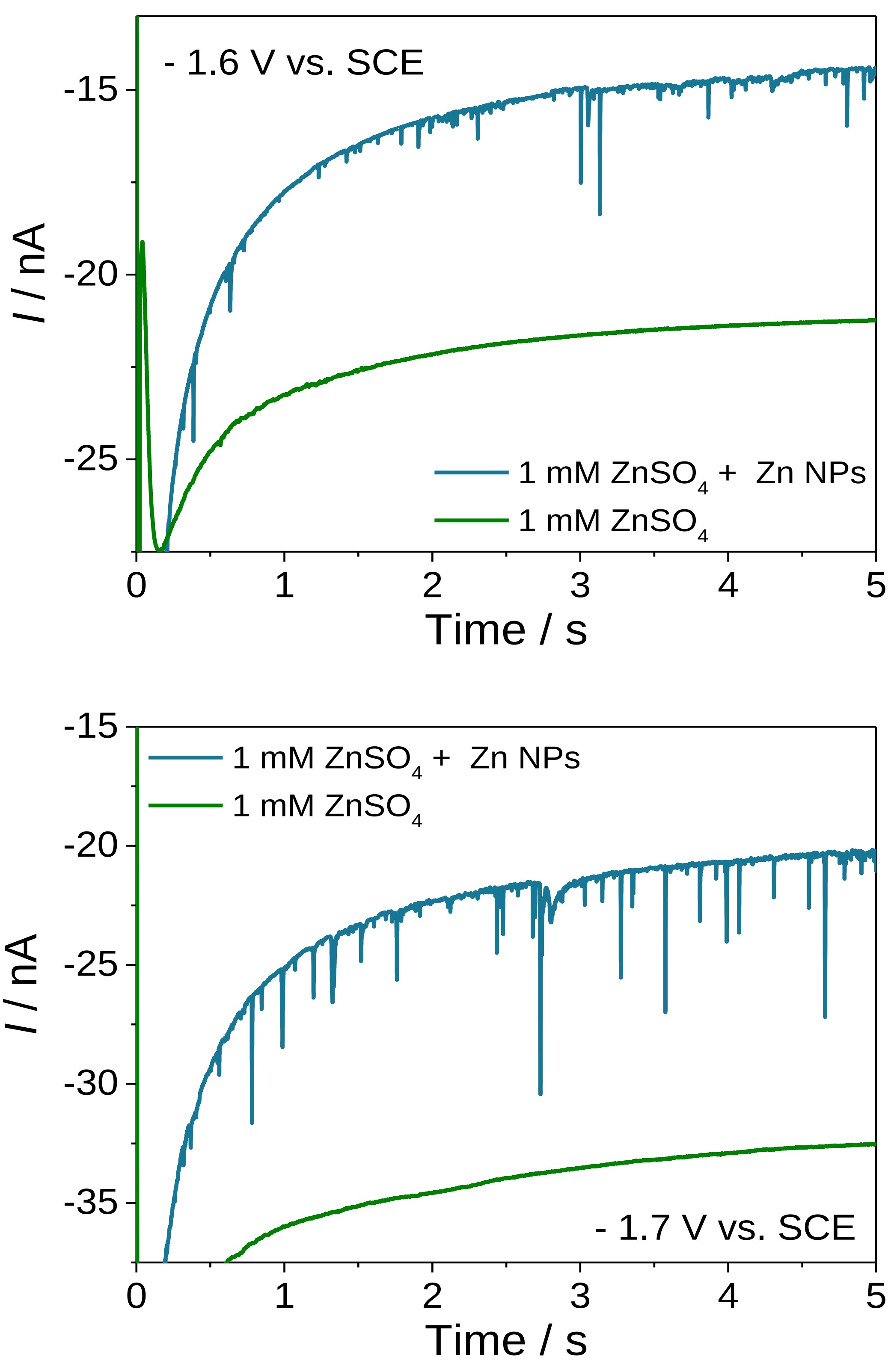


**Figure SI_1**: Chronoamperograms recorded at a carbon disk microelectrode immersed in a solution containing 1.0 mM $ZnSO_4$ with (blue line) and without 44 pM Zn nanoparticles (green line), at different applied potentials.

## 2. Control Chronoamperograms: with and without $Zn^{2+}$ ions in solution

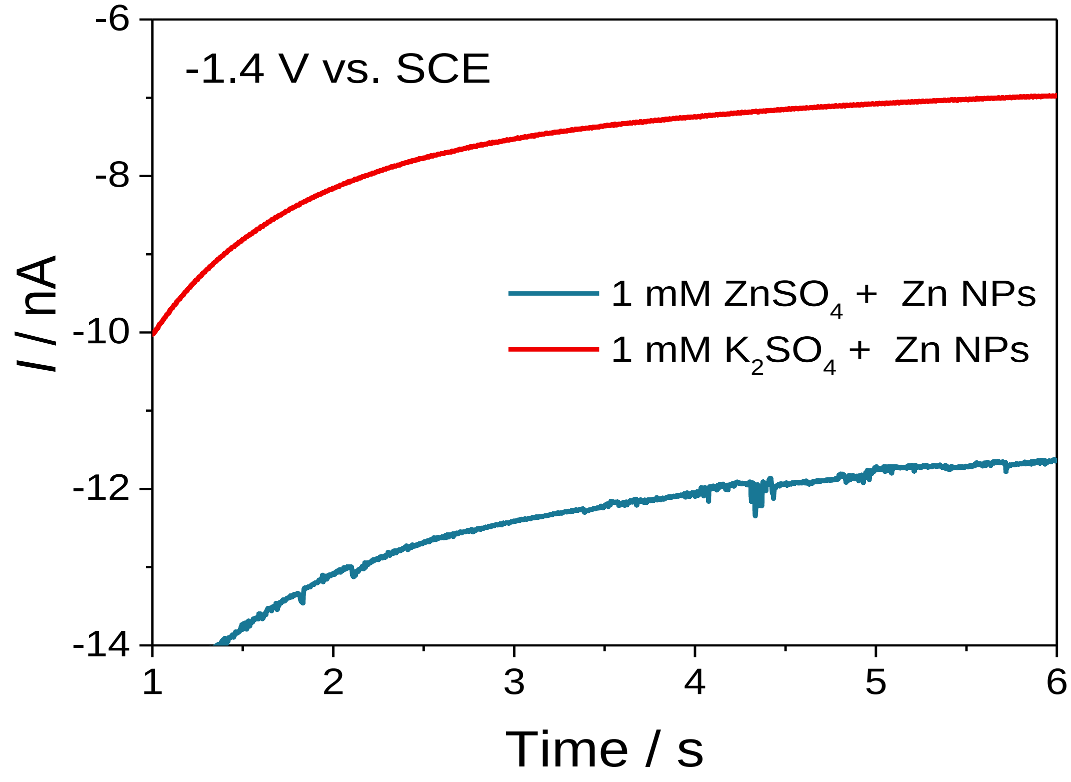


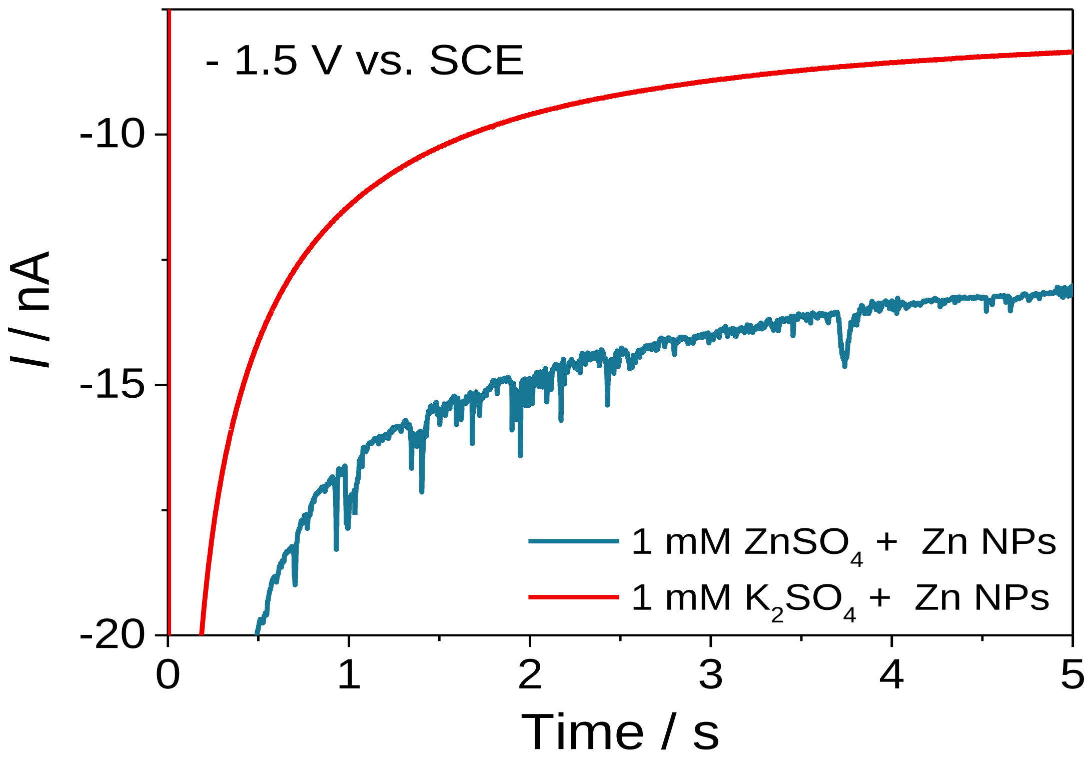

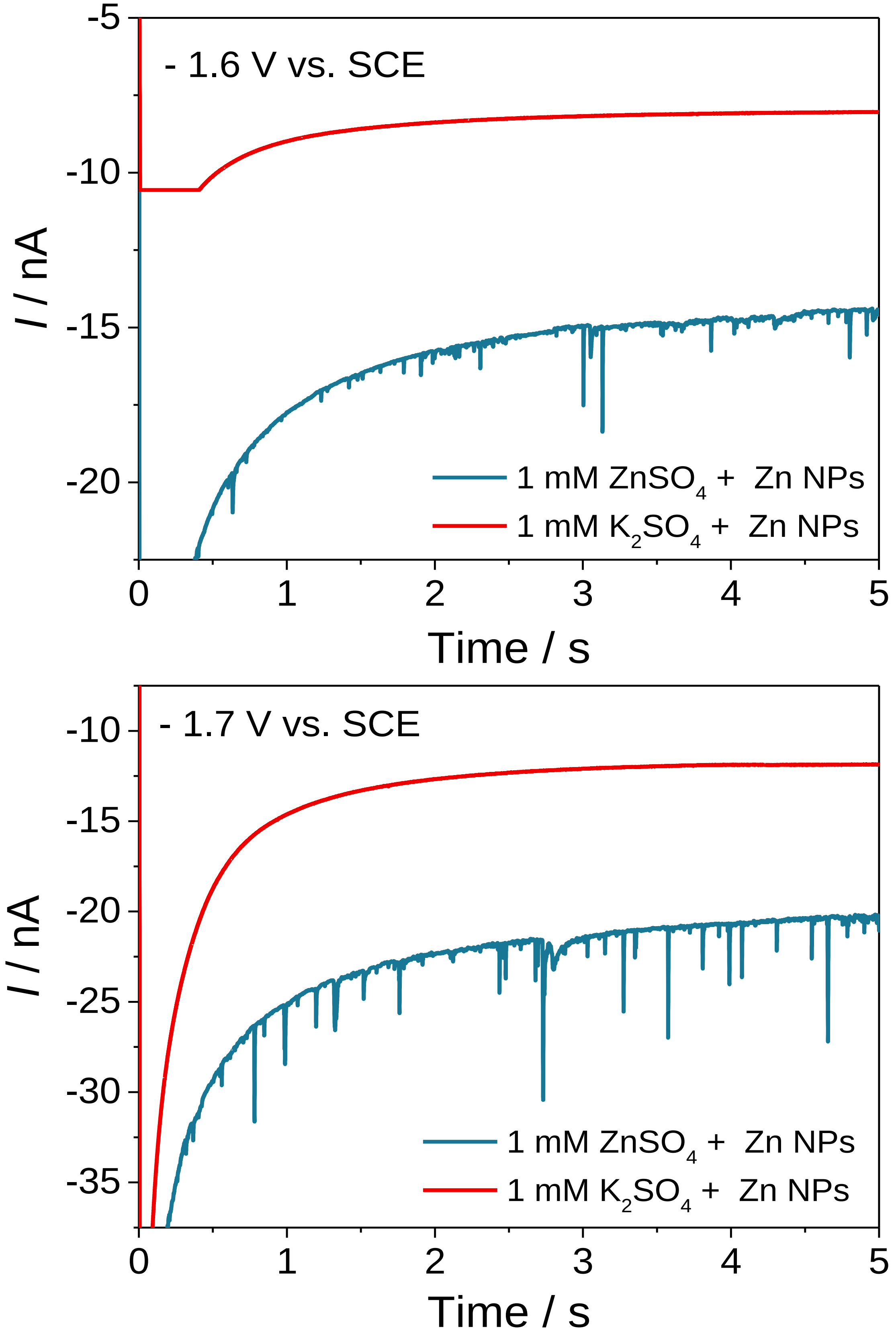


**Figure SI_2**: Chronoamperograms recorded at a carbon disk microelectrode immersed in a solution containing 44 pM Zn nanoparticles and 1.0 mM $ZnSO_4$ (blue line) or 1.0 mM $K_2SO_4$ (red line), at different applied potentials.

## 3. Control Chronoamperograms: Capacitive Current Contribution

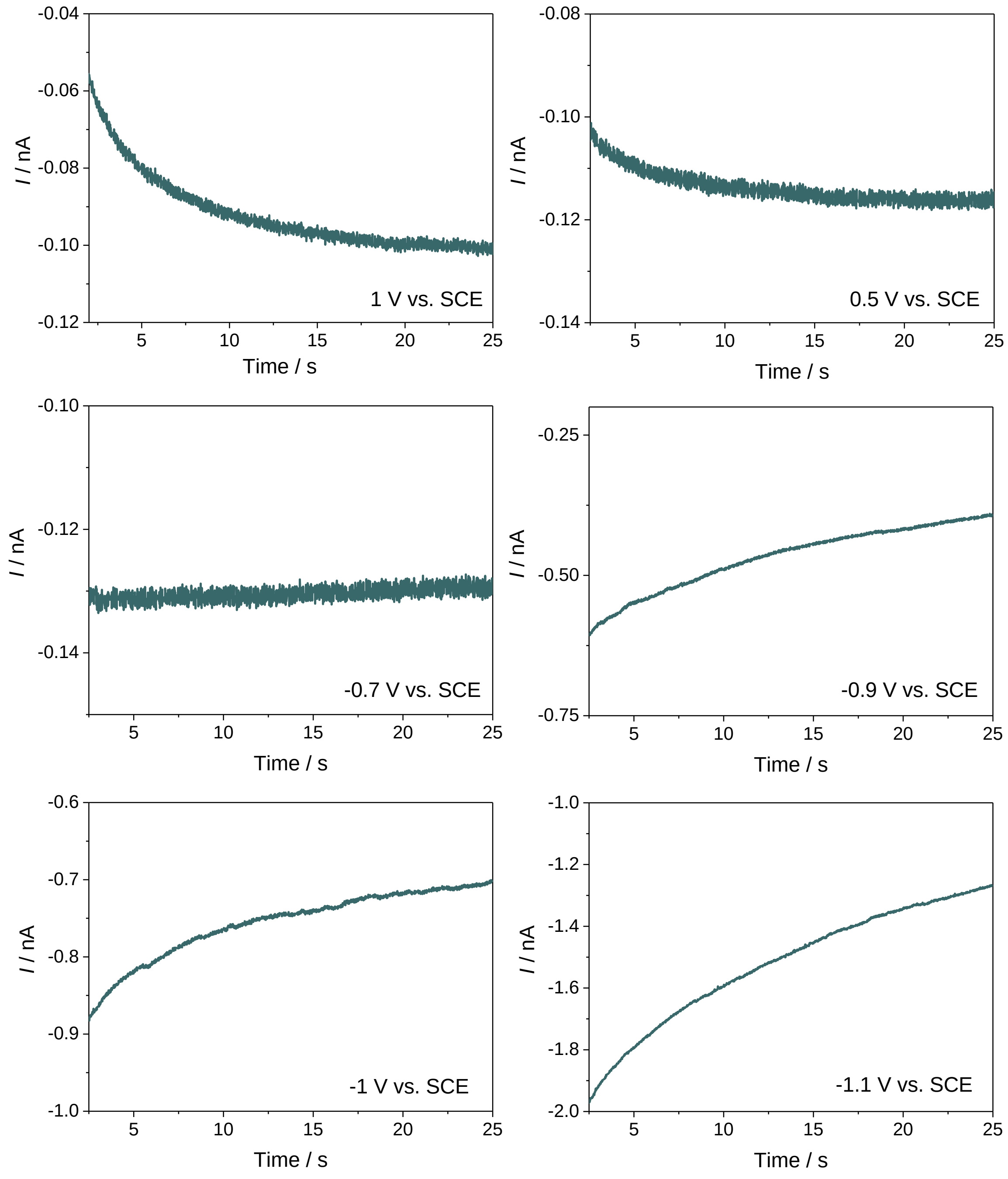


**Figure SI_3**: Chronoamperograms recorded at a carbon disk microelectrode immersed in a solution containing 44 pM Zn nanoparticles and 1.0 mM $ZnSO_4$, at different applied potentials

## 4. Zn Nanoparticles Scanning Electron Microscopy Imaging

a)

b)

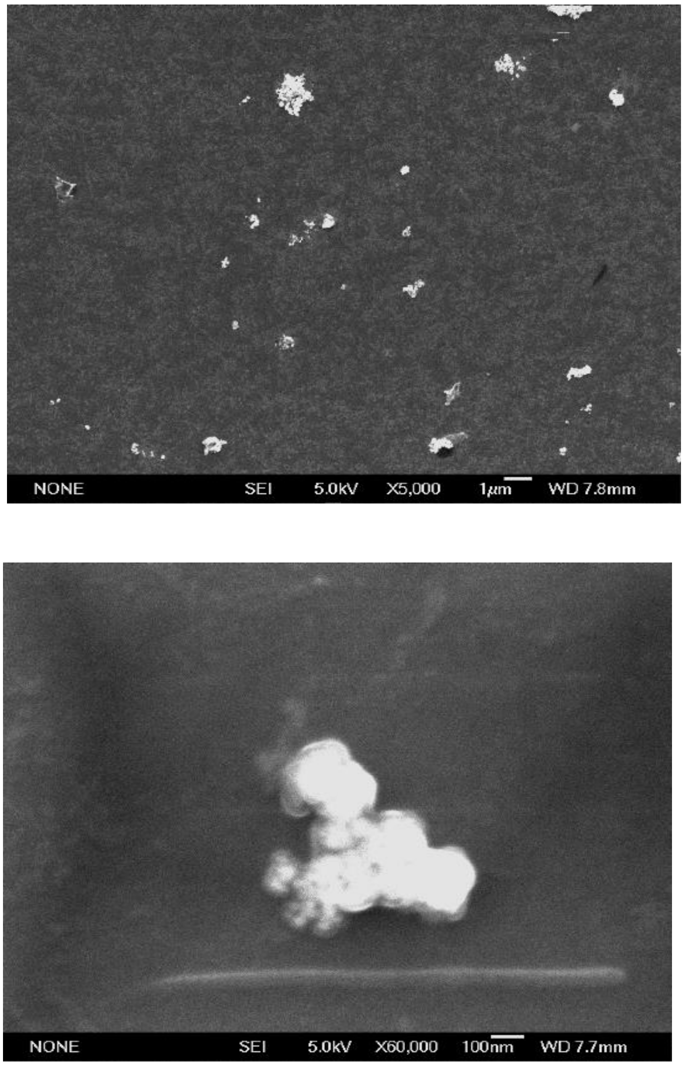


**Figure SI_4**: a), b) SEM images of Zn nanoparticles drop-cast onto glassy carbon electrode.

## 5. Average Collision Charge in 1.0 mM $ZnSO_4$ Solutions containing Different Zn Nanoparticles Concentration

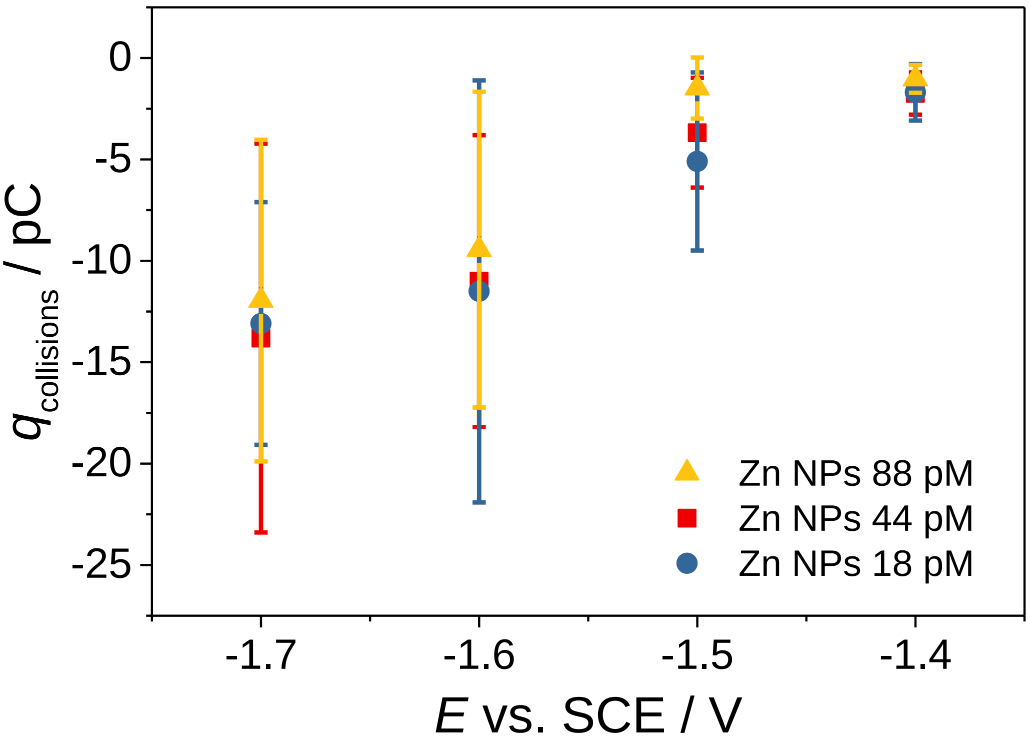


**Figure SI_5**: Average charge of the collision events (nanoimpacts) measured at different potentials applied at the carbon microelectrode in 1.0 mM $ZnSO_4$ solutions containing different amount of dispersed Zn nanoparticles.

## 6. Average Collision Duration in 1.0 mM $ZnSO_4$ Solutions containing Different Zn Nanoparticles Concentration

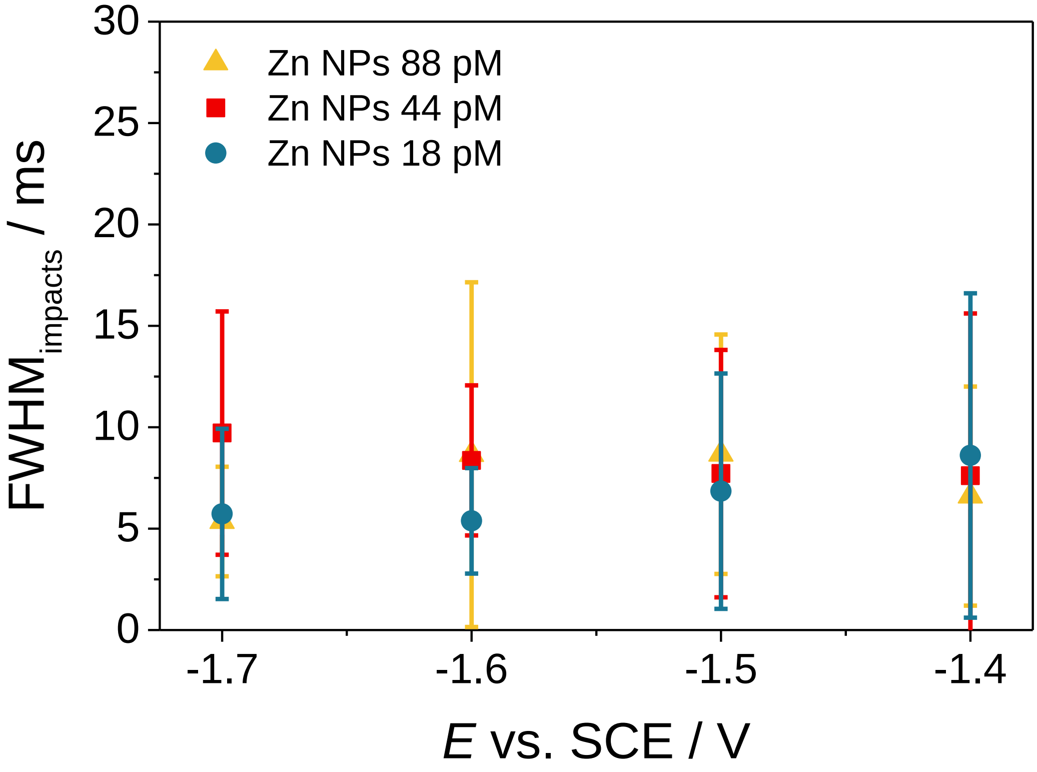


**Figure SI_6**: Average duration of the collision events (nanoimpacts) measured at different potentials applied at the carbon microelectrode in 1.0 mM $ZnSO_4$ solutions containing different amount of dispersed Zn nanoparticles.

## 7. Average Collision Frequency and Average Collision Duration in Supported and Unsupported Solutions

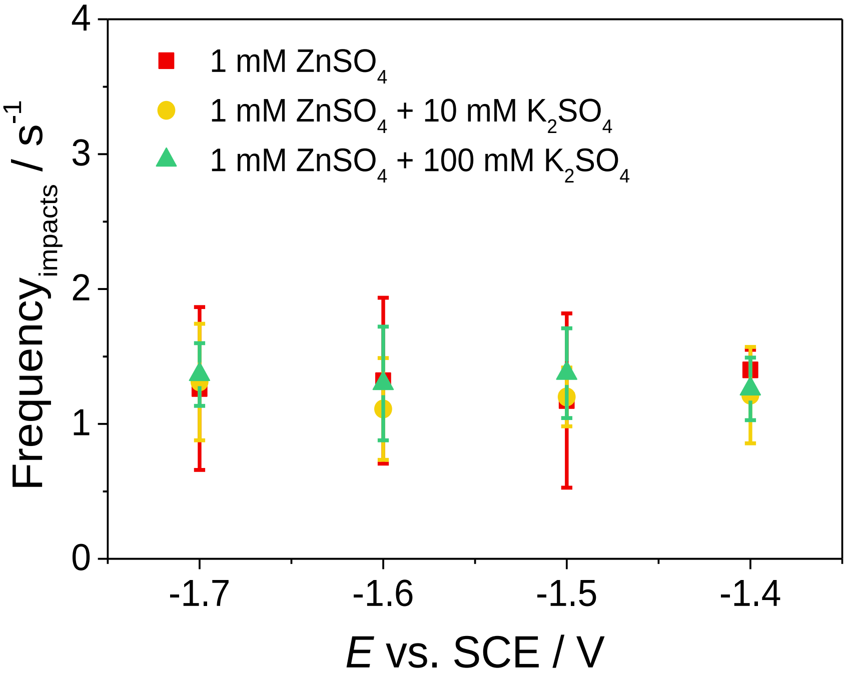


**Figure SI_7**: Average frequency of the collision events (nanoimpacts) measured at different potentials applied at the carbon microelectrode immersed in a solution containing 44 pM of dispersed Zn nanoparticles, with different electrolytes.

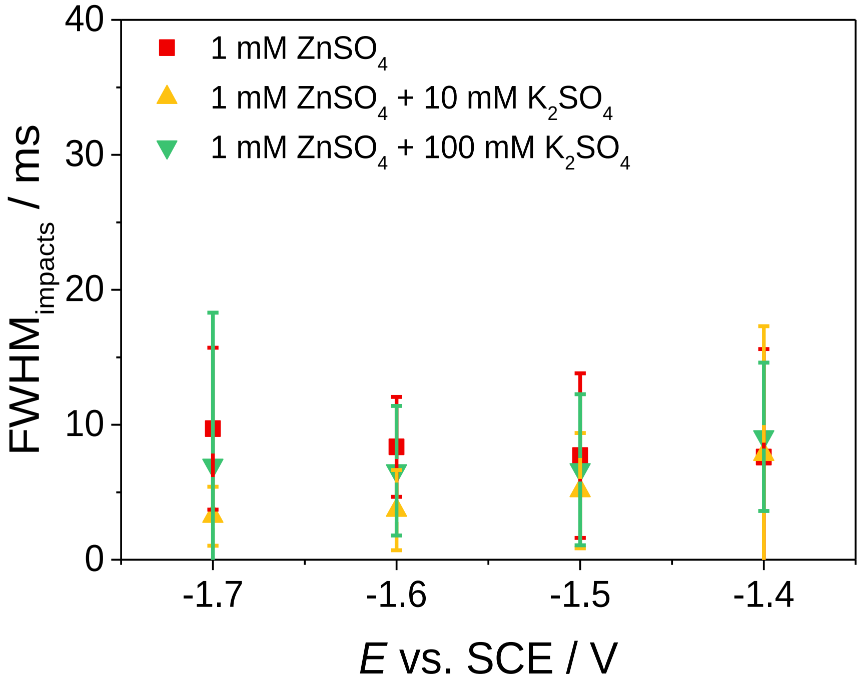


**Figure SI_8**: Average duration of the collision events (nanoimpacts) measured at different potentials applied at the carbon microelectrode immersed in a solution containing 44 pM of dispersed Zn nanoparticles, with different electrolytes.

## 8. Estimation of the Flux and Electrochemical Rate Constant

Considering the average current recorded during the impacts of the Zn NPs:

| Average Current Impacts – *I* [A] | | | |
|---|---|---|---|
| ***E* [V]** | **1 mM $ZnSO_4$** | **1 mM $ZnSO_4$ + 10 mM $K_2SO_4$** | **1 mM $ZnSO_4$ + 100 mM $K_2SO_4$** |
| -1.4 | $-9.86 \times 10^{-11}$ | $-1.52 \times 10^{-10}$ | $-1.04 \times 10^{-10}$ |
| -1.5 | $-3.69 \times 10^{-10}$ | $-4.89 \times 10^{-10}$ | $-6.81 \times 10^{-10}$ |
| -1.6 | $-1.81 \times 10^{-9}$ | $-5.27 \times 10^{-10}$ | $-8.61 \times 10^{-10}$ |
| -1.7 | $-2.06 \times 10^{-9}$ | $-1.22 \times 10^{-09}$ | $-2.83 \times 10^{-09}$ |

the dimension of the average Zn nanoparticle having:

NP average radius = 500 nm

NP average area = $1.9635 \times 10^{-09}$ cm$^2$

and the number of electrons involved in the Zn deposition reaction together with the Faraday constant:

n = 2

F = 96485 C mol$^{-1}$

the flux can be calculated according to :

$$J = \frac{I}{nFA}$$

| **Flux – *J* [mol cm$^{-2}$ s$^{-1}$]** | | | |
|---|---|---|---|
| ***E* [V]** | **1 mM $ZnSO_4$** | **1 mM $ZnSO_4$ + 10 mM $K_2SO_4$** | **1 mM $ZnSO_4$ + 100 mM $K_2SO_4$** |
| -1.4 | $2.60 \times 10^{-7}$ | $4.00 \times 10^{-7}$ | $3.68 \times 10^{-7}$ |
| -1.5 | $9.74 \times 10^{-7}$ | $1.29 \times 10^{-6}$ | $1.79 \times 10^{-6}$ |
| -1.6 | $4.79 \times 10^{-6}$ | $1.39 \times 10^{-6}$ | $2.27 \times 10^{-6}$ |
| -1.7 | $5.43 \times 10^{-6}$ | $3.22 \times 10^{-6}$ | $7.48 \times 10^{-6}$ |

Considering the following, one can estimate the rate constant for the Zn deposition reaction:

$|i_c| = nF\ K\ [Zn^{2+}]$ (A cm$^{-2}$)

$J = \frac{|i_c|}{nF} = K\ [Zn^{2+}]$ (mol cm$^{-2}$ s$^{-1}$)

$K = \frac{J}{[Zn^{2+}]}$ (cm$^{-1}$ s$^{-1}$)

$[Zn^{2+}] = 10^{-3}$ mol l$^{-1}$ = $10^{-6}$ mol cm$^{-3}$

| **Electrochemical rate constant – *K* [cm$^{-1}$ s$^{-1}$]** | | | |
|---|---|---|---|
| ***E* [V]** | **1 mM $ZnSO_4$** | **1 mM $ZnSO_4$ + 10 mM $K_2SO_4$** | **1 mM $ZnSO_4$ + 100 mM $K_2SO_4$** |
| -1.4 | 0.26016 | 0.39989 | 0.36854 |
| -1.5 | 0.97398 | 1.29183 | 1.79648 |
| -1.6 | 4.78863 | 1.39123 | 2.2733 |
| -1.7 | 5.43298 | 3.22254 | 7.48181 |
